# Minutes of the Twenty-First Annual Meeting of the Mississippi Valley Association of Dental Surgeons

**Published:** 1865

**Authors:** Will. Taft


### MINUTES OF THE TWENTY-FIRST ANNUAL MEETING OF THE MISSISSIPPI VALLEY ASSOCIATION OF DENTAL SURGEONS.

The Mississippi Valley Association of Dental Surgeons met pursuant to adjournment, February 22d, 1865, at half-past ten o'clock, in the lecture room of the Ohio Dental College. President A. Berry in the chair.

Minutes of the previous annual meeting read and approved.

The Secretary being absent, Wm. Taft was appointed Secretary *pro tem.*

Roll called. The following members were present: Drs. A. Berry, J. Taft, A. M. Leslie, Jas. Taylor, Chas. Bonsall, H. R. Smith, H. A. Smith, W. Taft, Chas. H. James, Sam. Wardle, Cincinnati; O. H. McCollum, Augusta, Ky.; J. W. Baxter, Warsaw, Ky.; J. P. Ulery, Rising Sun, Ind.; A. S. Talbert, Lexington, Ky.; Geo. W. Keeley, Oxford, O.; J. A. McClelland, W. H. Shadoan, Louisville, Ky.; H. A. Beamer, Cynthiana, Ky., and Julius Cheesebrough, Toledo, Ohio.

The Executive Committee submitted the following order of business.

1. Report of officers.
2. Election of officers to take place at ten A. M. to-morrow, the 23d.
3. Miscellaneous business. Reports of committees in order at any interval in discussions.
4. Essays, or papers to be read on the opening of discussions.

*Subjects for Discussion.*

1. Anæsthetics.
2. Filling teeth, material and instruments used.
3. Diseases of the teeth—Pathological conditions. Alveolar abscess. Sensitive dentine. Absorption and reproduc- of dental tissues.

4. Mechanical appliances. New inventions.

5. The true position of the dentist in relation to his patient, his student and his profession.

JULIUS CHEESEBROUGH, H. A. SMITH, A. S. TALBERT, } Com.

Drs. H. C. Howells, Hamilton, O., S. D. Tuttle, Eaton, O., J. G. Van Mater, Lyons, N. Y., J. C. Dean, Chicago, Ill., Geo. W. Gray, Albany, Oregon, A. A. Blount, Springfield, O., J. T. Johnson, Chattanooga, Tenn., George H. Cushing, Chicago, Ill., A. T. Metcalf, Kalamazoo, Mich., Wm. H. Sedgewick, Granville, O., W. P. Horton, Cleveland, O., R. A. Mollyneaux, New Richmond, O., J. C. Stephan, Cleveland, O., R. E. Taylor, Jas. I. Taylor, J. S. Waller, J. G. Cameron, Cy. M. Wright, Cincinnati, O., G. L. Hare, Covington, Ky., R. J. Poore, Lexington, Ky., H. H. Winne, Canton, Ill., F. Slayton, Florance, Italy, were elected members of the Association.

On motion, it was adopted that an investigating committee be appointed. Drs. Shadoan, Talbert and Cushing were appointed.

On motion, Dr. W. H. Shadoan, H. A. Smith and J. Taft were appointed a committee to procure the services of a reporter, with instructions to act immediately.

The committee to examine essays on anæsthetics and award the prize to the most deserving one, reported that no essays had been received, consequently no action.

On motion, adjourned to meet at two P. M. same day.

### AFTERNOON SESSION.

*February* 22*d.*—President Berry in the chair. Minutes of morning session read and approved.

The Committee appointed to secure a reporter, submitted the following:

The Committee on the procurement of a reporter would beg leave to report that they have secured the services of a reporter at $20 per day, the report to be prepared for the press.

W. H. SHADOAN,<br>H. A. SMITH,<br>J. TAFT, } Committee.

Their action was sanctioned, and the Committee discharged.

Dr. J. Taft read a communication from Dr. Pease, of Dayton, O., upon the subject of the renewal of the Goodyear rubber patent, which suggested that some action be taken upon it by the Association.

On motion, a committee of three, Drs. Cheesbrough, Horton and Shadoan were appointed to take the subject into consideration.

Fourth order of business. Dr. Cheesbrough read a paper entitled, "Anæsthetics, their properties, constituents, and modes of administering," for which a vote of thanks were tendered, and a copy for publication requested.

On motion, adjourned until to-morrow at nine A. M.

### MORNING SESSION.

President Berry in the chair.

Minutes of the previous session read and approved.

Committee on the Goodyear patent gave their report, and presented a written remonstrance for the reception of signatures. The Committee was continued.

The first subject (anæsthetics) was taken up and discussed.

The hour appointed for the election of officers having arrived, the association proceeded to ballot with the following result:

President, Dr. Julius Cheesebrough.

Vice President, Dr. J. A. McClelland.

Recording Secretary, Dr. Will. Taft.

Corresponding Secretary, Dr. H. A. Smith.

Treasurer, Dr. J. Taft.

Executive Committee, Drs. G. W. Keely, J. P. Ulrey, J. G. Van Marter.

Publication Committee, Drs. J. Taft, H. R. Smith, C. M. Wright.

The following gentlemen were appointed to prepare dissertations for the next meeting. Drs. Van Marter, J. C. Dean, C. M. Wright, A. S. Talbert and Geo. H. Cushing.

Drs. Berry, Shadoan and McClelland were appointed an Examining Committee.

The discussions were continued.

On motion, Drs. Shadoan, J. Taft and McClelland, were appointed a committee to make nominations for delegates to the American Dental Association.

On motion, adjourned to meet at two P. M.

### AFTERNOON SESSION.

*February* 23*d.*—President Cheesebrough in the chair, minutes of morning session read and approved.

A communication from Dr. S. S. Cobb was read, and on motion received.

The second subject for discussion was taken up.

The Committee to nominate delegates to the American Dental Association, reported as follows :

Dr. A. S. Talbert, Lexington, Ky ; Dr. W. H. Shadoan, Louisville, Ky. ; Dr. J. P. Ulrey, Rising Sun, Ind. ; Dr. H. C. Howells, Hamilton, O. ; Dr. Will. Taft, Cincinnati, O. ; Dr. C. H. James, Cincinnati, O. ; Dr. G. W. Keely, Oxford, O. ; Dr. W. H. Sedgewick, Granville, O. ; Dr. W. P. Horton, Cleveland, O. ; Dr. S. D. Tuttle, Eaton, O.

The above names of members of this Association are selected by this Committee as delegates to the American Dental Association.

J. TAFT,<br>

J. A. McCLELLAND, } Committee.

On motion, it was adopted that the report of the Commitmittee on the most valuable invention, discovery, or improvement, be made first in order at the evening session.

The Association adjourned until seven P. M.

### EVENING SESSION.

*February* 23*d*.—President Cheesebrough called the meeting to order at half-past seven P. M.

Minutes read and approved.

The Committee on the award of a medal for the best invention, &c., gave in the following report:

We of the Committee appointed to examine the merits of improvements in competion for award of silver medal, submit, that our attention has been called to the following named articles:

To-wit: Automatic plugger by J. C. Dean, Chicago, Ill.; ditto, by J. W. Baxter, Warsaw, Kentucky; also, by G. Jackson, of Winona, Minnesota. Buckeye Vulcanizer, by C. H. James, Cincinnati, Ohio. Foil crimper and cofferdam by J. A. McClelland, Louisville, Ky., and an annealing lamp by A. T. Metcalf, Kalamazoo, Mich. While we would commend the ingenuity and usefulness of all, yet for practical utility in supplying the wants of the profession, we believe the award should be made to C. H. James for improvement in vulcanizer, by which rubber packing is dispensed with, a ground joint being substituted and a single screw.

A. S. TALBERT,<br>W. H. SHADOAN, } Committee.

Which was accepted, and after considerable discussion, respecting the justness of awards being made to patented articles, was adopted.

On motion of Dr. Shadoan, it was adopted that the Associatien award a silver medal, the value of which shall be $20 for the best invention, discovery or improvement presented at the next annual meeting, and that a Committee of Three be appointed to decide upon the merits of said inventions, &c.

Drs. Talbert, Shadoan and Driggs were appointed the Committee.

On motion, Drs. Cy. M. Wright and H. A. Smith were appointed a committee to arrange a device for the medal to be given this year.

Dr. Cheesebrough deposited $10 for the purchase of a medal to be presented for the second best invention, &c. The award being subject to the same committee.

On motion, the Association tendered thanks to these gentlemen who had brought improvements before the Association.

Dr. H. A. Smith deposited the sum of $5 to procure a medal for the third best invention, discovery or improvement. Subject to the decision of the Committee on Awards.

The third subject for discussion was taken up. (Pathological conditions, &c.)

Dr. H. A. Smith read a paper upon the subject of periostites for which a vote of thanks was tendered, and a copy re quested for publication.

A communication was read from Dr. Driggs expressing regrets on account of unavoidable absence, and reporting a case.

A vote of thanks was tendered the Doctor; also a request for a copy to be published.

The subject in discussion was then continued.

The Association adjourned to meet to-morrow at nine A. M.

### MORNING SESSION.

*February 24th.*—The President in the chair. Minutes read and approved.

Dr. James Taylor made a motion to award a medal, value $25, for the best essay on alveolar abscess, which was adopted. The said essays to be examined by the Committee on Anæsthetics.

The following amendment was offered by Dr. Watt, that prize essays be copy-righted, the copy right to be the property of the Association.

Dr. Taylor offered the following:

"*Resolved,* That the Association award no medal or reward to patented articles of any description."

Dr. C. H. James asked if the medal awarded to him was intended to take the place of a patent.

Dr. Taylor sustained his resolution. The subject of which gave rise to *exciting* discussions between the believers and unbelievers. The resolution was finally adopted.

On motion, it was determined that when we adjourn it be to February, 1866.

On motion, a vote of thanks was tendered to the Cincinnati Dental Association for the entertainment given the Association.

"*Resolved*, That an appropriation of $15 be made and paid for janitor's services during the session of the Association."

The above was adopted.

The subject in discussion was continued.

The Treasurer made the following report which was received:

REPORT OF TREASURER, FEB. 24TH, 1865.

J. Taft, in account with the Mississippi Valley Association of Dental Surgeons:

| | |
|---|---|
| To Balance on hand at last report | $21 68 |
| To Annual Dues for 1864 | 19 00 |
| To Initiation Fees | 15 00 |
| | $55 68 |
| Cr. By amount paid Janitor last year | 8 00 |
| Balance in Treasury | $47 00 |

Respectfully submitted,

J. TAFT, Treasurer.

Drs. Talbert and Sedgewick were appointed a Committee to audit the above.

The next subject for discussion was taken up.

On motion, a vote of thanks was tendered Dr. J. A. McClelland for the presentation of appliances and descriptions thereof for correcting irregularities.

The next subject was taken up and discussed by Drs. Berry, Taft, Taylor and others.

The Auditing Committee reported as follows:

The Committee to audit the report of the Treasurer, find the same correct.

A. S. TALBERT, } Committee.

W. H. SEDGEWICK, }

"*Resolved,* That all dental and medical journals be requested to publish the proposition of this Association to award the various medals mentioned in the proceedings of the society."

"*Resolved,* That the President of this Association and members present at the next meeting of the American Dental Association have power to fill vacancies if any occur in the delegation."

Both of the above resolutions were adopted.

"*Resolved,* That the proceedings and discussions of this Association be published in the *Dental Register of the West.*"

On motion, the Association adjourned to meet on the third Wednesday in February, 1866.

WILL. TAFT, *Secretary.*

